# Autism in Preschool-Aged Children: The Effects of COVID-19 Lockdown

**DOI:** 10.1007/s10803-023-06078-4

**Published:** 2023-08-04

**Authors:** Cristiano Termine, Vera Galli, Linda Greta Dui, Valentina Berlusconi, Rossella Lipari, Francesca Lunardini, Simona Ferrante

**Affiliations:** 1 Child Neuropsychiatry Unit, Department of Medicine and Surgery, University of Insubria, Piazza Biroldi 19, 21100 Varese, Italy; 2 Department of Maternal and Child Health, Del Ponte Hospital, Varese, Italy; 3 Child Neuropsychiatry Unit, Department of Clinical and Experimental Sciences, University of Brescia, Brescia, Italy; 4 Department of Electronics, Information and Bioengineering, Politecnico Di Milano, Milan, Italy

**Keywords:** COVID-19, Autism spectrum disorder, Behaviour changes, Infant mental health, Lockdown

## Abstract

The COVID-19 lockdown affected children, especially those with autism spectrum disorder, due to the disruption in rehabilitation and educational activities. We conducted a cross-sectional study of 315 preschool-aged children, 35 of which had autism, to investigate this impact. A questionnaire was administered to explore socio-demographic status, familiar/home environment, and COVID-19 exposure. The clinical features of autistic subjects were also examined. Seven variables were considered to describe the effect of pandemic: *Remote learning*, *Behavior changes*, *Home activities*, *Sleep habits*, *Night awakenings*, *Physical activity*, *Information about the virus*. The lockdown had a significant impact on *Remote learning*, *Behavior changes*, and *Information about the virus* in participants with autism. Moreover, we found a worsening in repetitive movements, echolalia, restricted interests, and aggressive behaviors.

**Supplementary Information:**

The online version contains supplementary material available at 10.1007/s10803-023-06078-4.

## Introduction

The spread of COVID-19 disease in Italy began in February 2020, thus making the country one of the first victims of the pandemic. Government institutions faced an unknown threat for which there was no effective therapy or prevention, and the only solution was to interrupt all social activities. Even if some population segments were only marginally touched by the contagion, the sudden interruption of in-presence work and educational and therapeutic activities heavily impacted all people’s lives. This was particularly true for children, who suffered more from the lockdown measures than from the virus per se, at least during the first phase of the pandemic (Golberstein et al., 2020; Poletti & Raballo, 2021).

### Lockdown Impact on Preschool Children with ASD

While the COVID-19 lockdown was an unsettling period for most people, it was recognized to have been particularly hard for individuals with pre-existing learning, social, or behavioral difficulties, as is the case for neurodevelopmental disorders (Thorell et al., 2022; Guller et al., 2021). In an Italian study (Termine et al., 2021), the authors analysed more than 8000 questionnaires from school-aged children and adolescents with and without neurodevelopmental disorders and found that younger children suffered the most from the interruption of learning activities. This evidence was a prompt to focus on the impact of the pandemic on neurodevelopmental disorders in preschool-aged children. Indeed, preschool years represent a sensitive phase of development (Frankenhuis & Walasek, 2020; Woodar & Pollak, 2020), and integrated services in childcare (e.g., kindergartens, playrooms) are essential to providing affection, protection, and instruments through which to develop social, emotional, and cognitive abilities (Gordon et al., 2020; Mahoney et al., 2021). Discontinuation of rehabilitation therapies and closure of educational centers can block skill progress and lead to children with special needs missing the opportunity to advance their essential abilities (Lee, 2020).

Autism Spectrum Disorder (ASD) is one of the first neurodevelopmental disorders to emerge in children under 6 years of age (Persico, 2018); this may be due to the early manifestation of symptoms (e.g., motor stereotypies and eating and sleep issues) that impact families’ daily lives (Gabbay-Dizdar et al., 2022; Miller et al., 2021). Even if ASD is caused by genetic (Bailey et al., 1995; Muhle et al., 2004; Thapar & Rutter, 2021) and environmental factors (Emberti Gialloreti et al., 2019), stressful life events and routine/lifestyle changes are known to strongly contribute to the varied expression of autism-related traits (Fuld, 2018; Taylor & Gotham, 2016), and the pandemic certainly classifies as a stressful period. Some of the most relevant modifications in environment caused by this event were (1) the introduction of remote learning, (2) possible behavior changes due to routine alteration, (3) the shift from school to home activities, (4) the disruption of sleep habits, especially (5) night awakenings, (6) the potential interruption of physical activity due to home confinement, and (7) the increase in stress levels associated with an unknown virus. Such substantial changes in everyday life are added to the pathological traits that are characteristic of the ASD condition, such as inflexibility and trouble with variation in schedule (Reicher, 2020), with a potential regression of previously acquired socio-cognitive skills (Pearson et al., 2018). As for behavior, ASD-related aggressiveness and stereotypies were worsened by social deprivation (Feige et al., 2021) and changes in routine (Alhuzimi, 2021). Regarding home confinement, parents working from home, owing to a better work-life balance (Ipsen et al., 2021), could support ASD children in mitigating their symptoms (Tokatly Latzer et al., 2021; Zwaigenbaum et al., 2015); however, other studies suggest that the positive effect deteriorated when the lockdown lingered, which increased parents’ stress levels (Bozkus-Genc & Sani-Bozkurt, 2022). Sleep habits are also important to investigate, with insomnia and other sleep disturbances manifested by up to 80% of children with ASD between the ages of 4 and 6 years (Posar & Visconti, 2020). Indeed, other studies on the effect of the pandemic on sleep habits, which also included older children, reported that children with ASD had reduced sleep duration, anxiety at bedtime, and difficulties in falling asleep (Bruni et al., 2021). The interruption of physical activity hindered these positive benefits, such as, equilibrium between aggressive behaviors and self-control, tolerance of stress, improvement to executive functions, working memory, and communicative aspects (Bidzam-Bluma & Lipowska, 2018). Regarding virus-related stress, children with ASD may show limited interest in external events due to an avoidance attitude (O’Nions et al., 2018) or constant requests due to typically repetitive behavior.

Although other studies have investigated the effect of COVID-19 on these aspects, they usually focus on older ages, e.g., with regard to remote learning or home activities (Bozkus-Genc & Sani-Bozkurt, 2022), or they focus on single aspects such as sleep disturbance (Bruni et al., 2021) or behavior (Boterberg et al., 2022; Logrieco et al., 2022) rather than providing a comprehensive view, thus making it useful to further explore this topic. Lastly, the clinical implications of the COVID-19 lockdown should also be considered concerning the effect of the interruption of specific therapeutic plans, which probably caused at least the partial loss of the adaptive-relational expertise.

### Aim of the Study

The aim of this study is to analyze the psychological and environmental impact of the COVID-19 pandemic on a pre-school population through an online questionnaire completed by parents. We hypothesized that both socio-demographic and clinical variables can influence the seven identified environmental factors that were modified by the pandemic. More specifically, our aim was to investigate whether the ASD condition accounts for certain differences in these factors compared to a matched control group. Moreover, we also inspected the trend of the core symptoms of the ASD group during the two-month complete lockdown period that occurred unexpectedly.

## Methods

### Participants

Children from 3 to 6 years of age participated in this study. In Italy, children between 3 and 6 usually attend three years of kindergarten (i.e., preschool classes), which prepares them for primary school by helping them acquire basic learning skills and relational abilities. All participants were residents of Varese, an Italian province with high territorial homogeneity, mainly composed of medium-sized cities. The sample comprised both children with ASD and typically developing children who did not present either ASD or any other neuropsychiatric diagnosis. We leveraged different recruitment sources for subjects affected by ASD to enrich our sample. Hence, parents were asked to fill out an online questionnaire by the Child Neuropsychiatry Unit of Filippo Del Ponte Hospital in Varese and the ANGSA association (National Association of Autistic Subjects’ Parents, Associazione Nazionale Genitori Soggetti Autistici). Controls were recruited through the websites of kindergartens of the Province of Varese, although children with ASD from this source were also included. Specifically, families were alerted about the availability of questionnaires to be completed through email by kindergarten teachers and could access the questionnaires through direct links. For both recruitment sources (hospital/association and schools), the questionnaire was proposed to parents of all children to avoid any selection bias.

Data were collected from April to June 2020. This time frame included both the lockdown period (March–May 2020), when people were allowed to leave home only for urgent reasons, and the period immediately afterwards, when people were allowed to go to work if remote working was not an option. During this second phase, schools and rehabilitation services remained closed, making it comparable to the lockdown phase from the children’s perspective. Due to the young age of the participants, the questionnaires were filled in by parents only. Parents provided consent electronically by answering a direct question, wherein they agreed to the anonymous and confidential participation in the study (Supplementary Material 1, question 1), as per the privacy policy established by the Ethics Committee of ASST dei Sette Laghi di Varese (n.82 of 2020). The questionnaire was completely anonymous as was all the data provided, which was sent and collected in an anonymized form. By completing the questionnaire, the parent declared to have understood the purposes of the study and agreed to participate by providing the anonymous data necessary for its implementation. The study was conducted in compliance with the Declaration of Helsinki.

### Questionnaire Design

An online questionnaire (Supplementary Material 1) was designed by a multidisciplinary team of psychologists and child neuropsychiatrists. The first section (questions 2–16) included questions that were useful for characterizing the sample by exploring the demographic and environmental features of the children and their families. First, children were stratified according to ASD or typical development condition. Given the heterogeneity of the recruitment and no possibility of a clinical investigation, we were not able to assign a severity degree to the children with ASD included in the study. For this reason, all children with ASD were treated as a single group, against the typically developing children. We further stratified subjects according to age and gender, as the gender is known to have an association with the ASD condition, with greater prevalence in the male population (Loomes et al., 2017), at least during childhood (Posserud et al., 2021). We then considered the environmental factors that could have influenced lockdown management, i.e. socio-economic status (Hollingshead index, H-SES) (Hollingshead, 1975); the presence of parents at home—considering either family status (child living with one or both parents) and whether they worked from home due to the lockdown— or the presence of siblings that shared home resources but might contribute to engaging in activities with the toddler. The presence of an outdoor space was also investigated as it is known to have several benefits, such as orienting the plays in a spontaneous and creative direction (Lee et al., 2021) and promoting attention restoration and memory while moderating externalising behaviors (McKormick, 2017). Finally, we collected variables that we hypothesized could model the effect of the pandemic-induced anxiety, such as exposure to COVID-19, both in the family or outside the family, or parents working as healthcare professionals. The encoding of these variables is presented in Table 1.

**Table 1:** Answers describing the population: independent variables in a multi-variate model

2-Levels questions	0	1
Certified as ASD (question 4)	Control	ASD
Gender (question 3)	Male	Female
Family status (question 10)	Single-parent family/parents living separated	Parents living together
COVID-19 cases in the family (question 13)	No	Yes
Exposure to COVID-19 cases outside the family (question 14)	No	Yes
Outdoor spaces at home (question 12)	No	Yes

3-Levels questions	0	0.5	1
School class (question 5)	1st	2nd	3rd
Presence of siblings (question 11)	None	One	More than one
Parents working as healthcare professionals (question 15)	None	One	Both
Parents working from home (question 16)	None	One	Both

5-Levels questions	0	0.25	0.5	0.75	1
H-SES (question 6 to 9)	Very low	Low	Medium	High	Very high

**Fig. 1:**
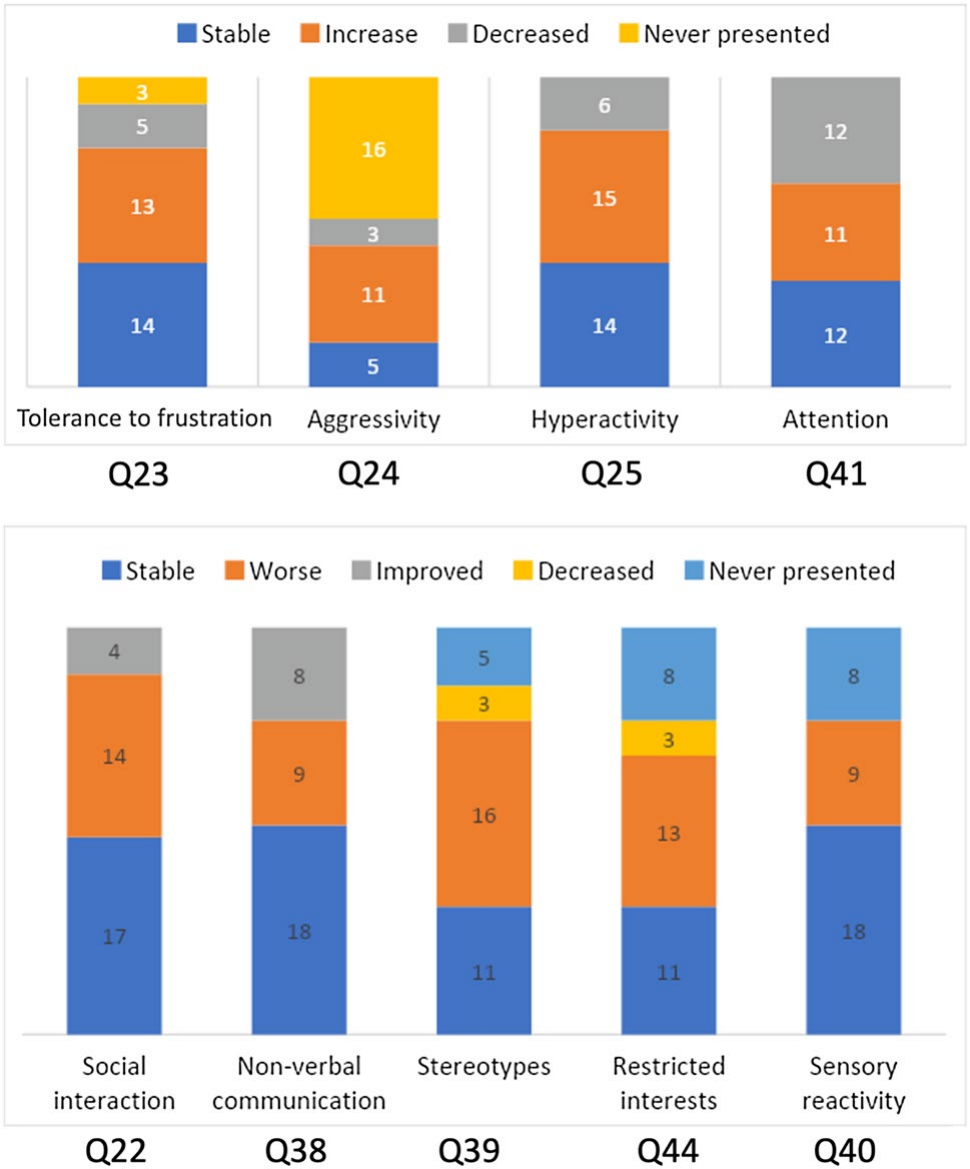
Alterations of clinical features in subjects with ASD; the numbers reported below the bars refer to the questions (see the questionnaire in Supplementary Material 1)

**Fig. 2:**
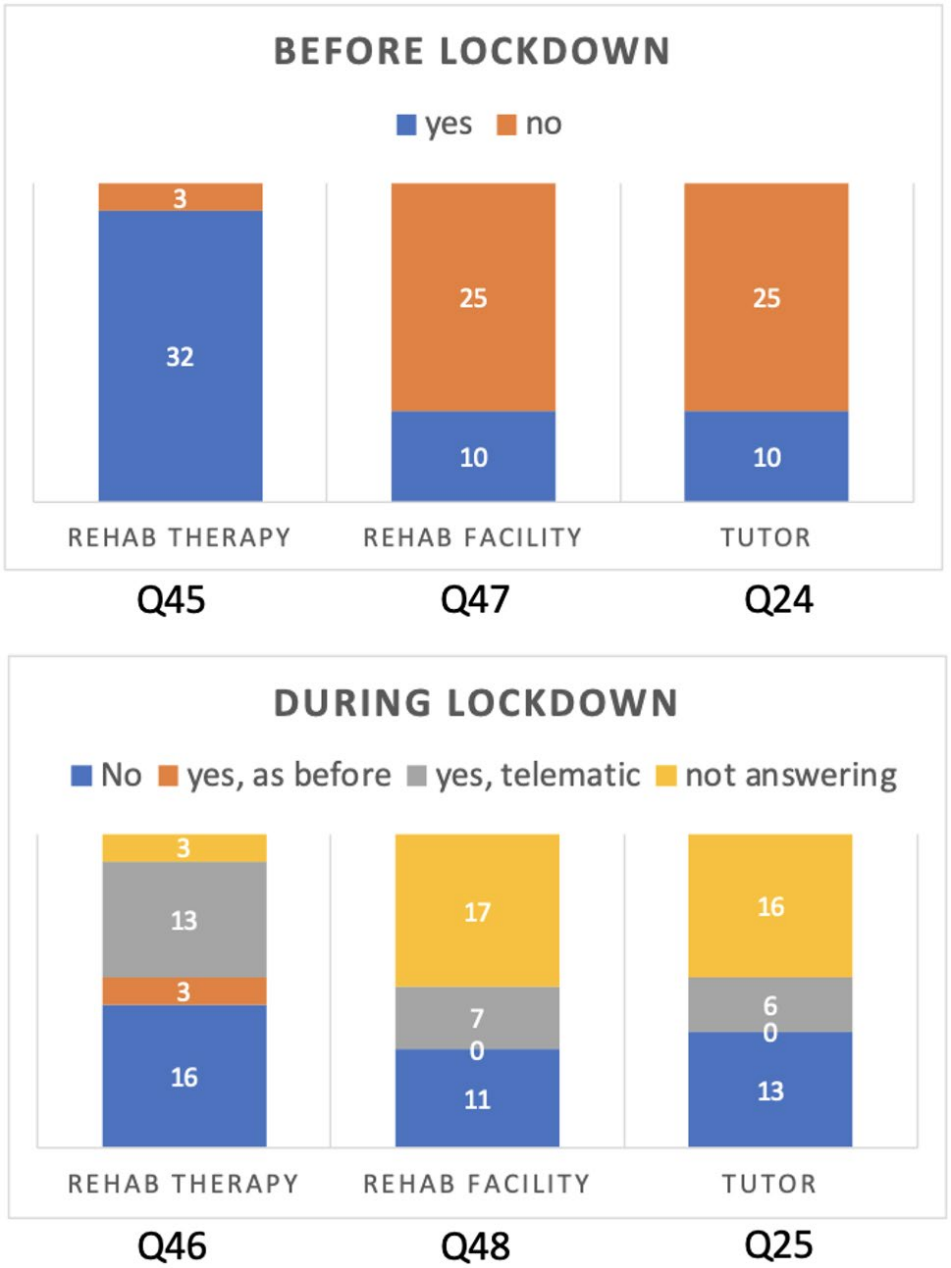
Changing in non-pharmacological therapy; the numbers reported below the bars refer to the questions (see the questionnaire in Supplementary Material 1)

After the characterization section, we proposed questions related to the different areas of interest (questions 17–30), as presented in Table 2. *Remote learning* was aimed at modelling whether kindergarten activities continued to be performed during the lockdown. *Behavior changes* described potential degeneration due to home confinement. *Home activities* was aimed at describing whether parents proposed alternative activities to substitute the lack of kindergarten. *Physical activity* inquired about the possibility of maintaining a healthy lifestyle during the lockdown. *Night awakening* and *sleep habits* investigated potential problems at night time. *Info virus* was mainly related to the fear that COVID-19 could have infected toddlers. A last section (questions 31–50) was targeted at participants affected by ASD only. Some of the questions were specifically aimed at investigating the ASD condition: *Irritability, Aggressivity, Hyperactivity, Attention, Social interaction, Non-verbal communication, Stereotypies, Restricted interests* and *Sensory reactivity* (Fig. 1). Whilst *Attention*, *Tolerance of frustration*, *Social interaction* and *Non-verbal communication* were considered negative outcomes if they showed a decrease, the other clinical features were considered negative outcomes if they showed an increase. Other questions were aimed at investigating drug therapy and non-pharmacologic support to children before the pandemic (Fig. 2).

**Table 2:** Answers intended to build dependent variables

Dependent Variables	Items to measure the variable	Answer encoding (0 = negative; 1 = positive)	Meaning and Direction
Remote learning	Attending remote educational activities (question 19)	No/With difficulties: 0 Yes: 1	Was it possible to follow remote educational activities/tutoring? How much did children miss school? *High values: well*
	Tutoring support (question 17–18)	Interrupted: 0 Unchanged: 1	
	Asking about the school reopening (question 20)	No: 0; Yes: 1	
Behaviour changes	General behaviour (question 21)	Worsened: 0; Stable/improved: 1	How do you judge the behaviour during lockdown? *High values: well*
	Social interaction (question 22)	Worsened: 0; Stable/Improved: 1	
	Tolerance to frustration (question 23)	Increased frustration: 0 Improved/stable/never presented: 1	
	Aggressiveness (question 24)	Increased aggressiveness: 0; Improved/stable/never presented: 1	
	Hyperactivity (question 25)	Increased hyperactivity: 0; Improved/stable/never presented: 1	
Home activities	Home activities to spend time together during lockdown (question 26)	No: 0; Yes: 1	Have you organised board games, handcrafts, cooking? *High values: yes*
Physical activity	Physical activity during lockdown (question 27)	Interrupted/diminished/never done: 0 Unchanged: 1	How did physical activity change? *High values: unchanged*
Sleep habits	Sleep–wake rhythm during lockdown (question 28)	Changed: 0; Unchanged: 1	Has sleep changed? *High values: No*
Night awakenings	Nightly awakenings during lockdown (question 29)	Increased: 0 Never presented/Unchanged/diminished: 1	Have nightly awakenings changed? *High values: unchanged or decreased*
Information about virus	Information request about COVID-19 (question 30)	Never/always: 0; Sometimes: 1	Did your child often ask about the coronavirus? *High values: sometimes*

### Data Analysis

We conducted a cross-sectional study. Given the small sample of children with ASD compared to the typically developing ones and to provide a meaningful sample, we reduced the number of typically developing children. Subsampling was performed to achieve a sample of Controls that was matched by age, gender, and socio-economic status. The proportion of ASD children was set to 10% and kept constant in the three variables used for matching the sample. As for the other variables that characterized the sample, we performed Fisher’s exact tests for categorical variables or Mann–Whitney U tests for ordinal variables (dependent variables) to describe any difference in the distribution between ASD and Controls (ASD condition was set as the independent variable).

Statistical analysis was conducted using R (version 4.0.1), with 0.05 considered as the significance threshold. Answers that characterized the population (Table 1) were included as independent variables in a multivariate model. Dependent variables were encoded assigning a positive (1) or negative (0) meaning, according to the latent trait modelled in each of the seven dependent variables (Table 2). When a single answer built the dependent variable (*Home activities; Physical activity; Sleep; Night awakenings; Information about virus*), it was kept as binary. Otherwise, when more than one answer built the dependent variable (*Remote Learning; Behavior*), the latent trait was achieved by applying a 2-parameter Item Response Theory model (IRT) (Hambleton & Swaminathan, 2013). To keep a meaning of low-medium–high value in the latent trait, whenever a higher granularity resulted from IRT, we chose the 33rd and the 66th percentiles to re-code it. To understand the effect of health, family, and environment (independent variables) on the dependent variables, we applied binary logistic regression or ordinal logistic regression, according to the granularity of the endpoint. After checking the assumptions of absence of multicollinearity between independent variables (variance inflation factors below 10) (Hair et al., 1998) and proportional odds for the ordinal dependent variables (Brant test) (Brant, 1990), we leveraged the Akaike Information Criterion to keep only the most significant covariates, according to both a forward and backward procedure. Then, only the significant independent variables were used in the final logistic regression models. In case the condition of being affected by ASD was eliminated during the Brant test analysis, two separate models (one for ASD cases and one for Controls) were built. To describe the clinical characteristics of the ASD sample, we applied frequency analysis on the remaining questions.

## Results

A total of 964 questionnaires were analyzed comprising 501 males and 463 females, 284 from the first year of kindergarten (median age: 4; interquartile range: 0), 342 from the second (age: 5; 0), and 338 from the third (age: 6; 0). Subjects with ASD were 35, which represents 3.6% of the sample. The original sample accounted for significantly more males in the ASD group (85.7%) than in typically developing children (50.7%), according to Fisher’s exact test (p < 0.001). ASD children were found predominantly in higher years of education (median and quartiles: 3 [2; 3]) than children from the typically developing group (2 [1; 3]), according to a Mann–Whitney U test (p = 0.010); and in lower socio-economic classes (ASD: 2 [1.5; 3.5]; comparison: 3 [2; 4]; p = 0.016).

**Fig. 3:**
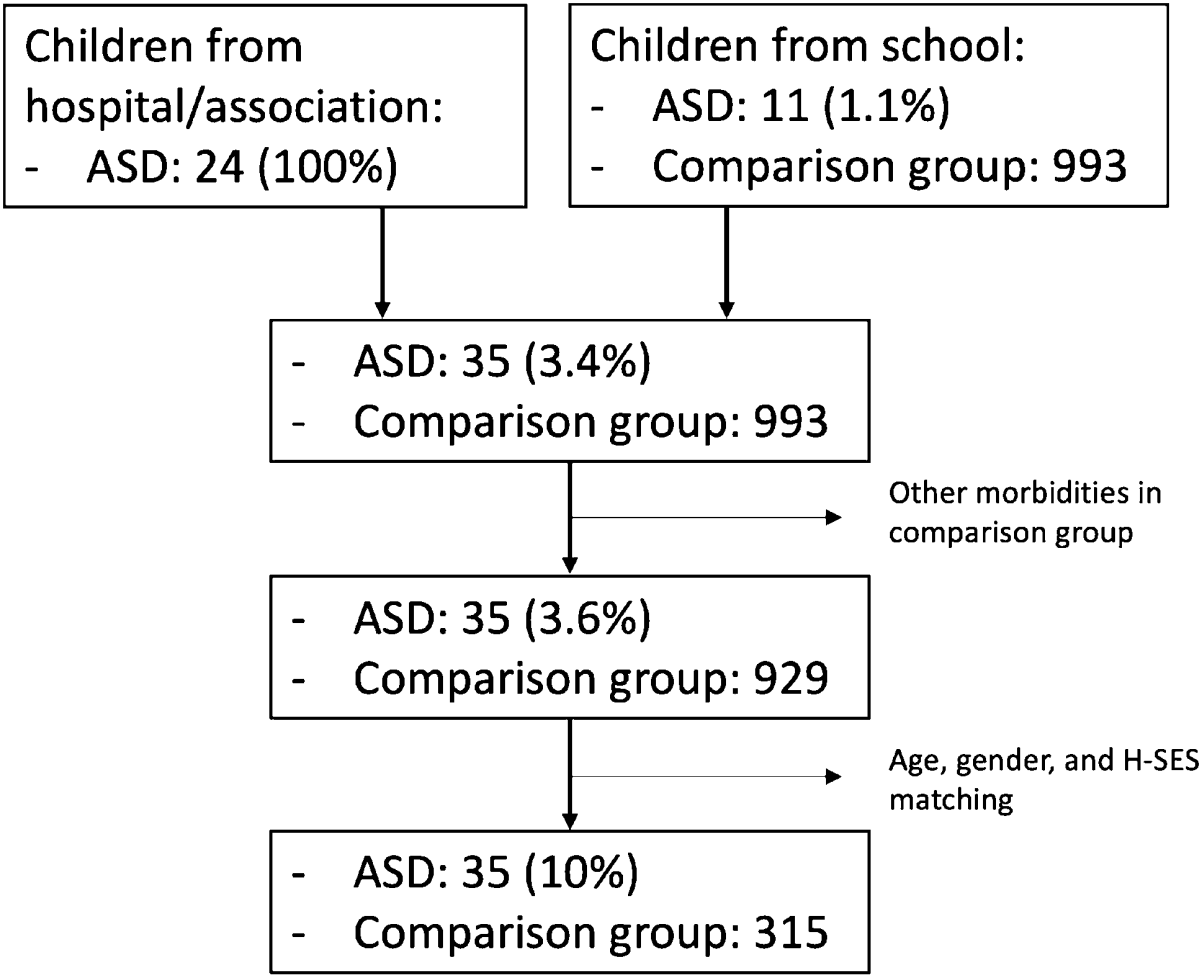
Flowchart for participants’ selection

After age, gender, and H-SES matching, a total of 350 questionnaires were retained, with 35 (10%) children with ASD and 315 Controls. A description of the selection process is provided in Fig. 3. A description of the population is presented in Table 3, where subjects are stratified by ASD or Control condition for each variable. Specifically, 300 males and 50 females were retained, 40 from the first year of kindergarten, 139 from the second, and 171 from the third. Aside from variables leveraged to match the sample, the ASD condition incidence was the same for the *family status* variable (p = 0 0.949), the *outdoor space* availability (p = 0.072), *COVID-19 cases inside family* (p = 0.378) and *COVID-19 exposure* (p = 0.715), nor did we found any significant difference between the distribution of the number of *siblings* (p = 0.205), *parents in remote working* (p = 1), and parents working as *health professionals* (p = 1) between the groups.

**Table 3:** Answer frequencies of the independent variables divided by Controls and ASD

Independent Variables	Controls	ASD
Gender		
Male	270	30
Female	45	5
H-SES		
1	54	9
2	86	10
3	66	7
4	82	6
5	27	3
Family status		
Divorced or single-parent	15	1
Parents living together	300	34
Siblings		
0	87	11
1	43	12
More than 1	185	12
Outdoor spaces		
No	42	9
Yes	273	26
School class		
First class	36	4
Second class	126	13
Third class	153	18
Parents in remote working		
None	195	22
One	75	8
Both	45	5
Health professional parents		
None	280	33
One	29	1
Both	6	1
COVID-19 cases inside family		
No	302	35
Yes	13	0
COVID-19 exposure		
No	293	34
Yes	22	1

### Logistic Regression

Results of the logistic regression are summarized in Table 4. The algebraic sign of the value represents the direction of the effect over the dependent variable, whereas the absolute value represents its importance. As shown, the presence of ASD had a strong negative effect on the *Remote learning* variable, suggesting that the presence of ASD made it more difficult to carry out remote education activities than in the control group. Male gender, high SES, and parents working as healthcare professionals are factors that negatively impacted on *Remote learning.* A significant negative effect of the ASD condition was also reported for the *Info-virus* variable, suggesting that children with ASD did not properly ask for information about the virus (asking too little or too much) compared to the control group. This was noted in subjects who were exposed to COVID-19 inside their families and those who could not play outside as well. As in the *Info-virus* variable, we considered both asking too frequently and never asking to be negative, and we further investigated whether the result was imbalanced towards one of the two extremes. A Fisher’s exact test revealed that a significant difference occurred between ASD and Controls on the three answers (never asking, sometimes asking, frequently asking, p < 0.001): 24 children with ASD never asked about the virus (68.6%, in contrast with 13.7% of the Controls, p < 0.001 in the Bonferroni-corrected post hoc), 2 ASD children frequently asked about the virus, but there was not a significant difference with Controls (ASD: 5.7%, Controls: 23.2%, p = 0.304), 9 ASD children sometimes asked about the virus (25.7%, in contrast with 63.2% of Controls, p < 0.001). Data show that the majority of children in both groups had fun activities in their families (334 positive answers), even if none of the variables had a significant impact on it; in both groups there were variations in the sleep–wake rhythm, with differences in sleeping and waking times compared to the period before the lockdown (it occurred for 262 children), and in night awakenings that decreased or remained stable in both groups (301 positive answers), but the situation was improved by the presence of an outdoor space to play in and in families where parents could not work from home. With regard to *Physical activity*, 302 of the children from both groups interrupted or reduced the frequency of activities. Finally, the *Behavior* variable differed so widely between the ASD and control group that it was necessary to build separate models. Having an outdoor space positively influenced the *Behavior* variable for the Controls: those who had an outdoor space available showed better behavioral modulation.

**Table 4:** Impact of independent variables on dependent variables

Remote learning			
Ordered significant covariates	value ± standard error	t-value	p-value
** ASD**	**− 2.376 ± 0.362**	**− 6.561**	** < 0.001**
	**ASD negatively impact Remote learning**		
** Health professionals**	**− 1.365 ± 0.556**	**− 2.453**	**0.014**
	**Parents working in health professions negatively impacted Remote learning**		
** H-SES**	**− 1.327 ± 0.420**	**− 3.156**	**0.002**
	**High H-SES negatively impacted Remote learning**		
** Sex**	**0.744 ± 0.298**	**2.494**	**0.013**
	**Remote learning is easier for females**		
Behaviour Changes (CONTROLS)			
Order Significant Covariates	value ± standard error	t-value	p-value
School class	**− **0.860 ± 0.462	**− **1.862	0.063
**Outdoor space**	**0.756 ± 0.293**	**2.577**	**0.010**
	**Outdoor space is associated with better behaviour in controls**		
Behaviour Changes (ASD)			
Order Significant Covariates	value ± standard error	t-value	p-value
H-SES	**− **2.575 ± 1.653	**− **1.558	0.119
Home working	1.833 ± 1.240	1.478	0.139
Home Activities			
Order Significant Covariates	value ± standard error	t-value	p-value
COVID-19 exposure	**− **15.599 ± 1360.064	**− **0.012	0.991
Sleep Habits			
Order Significant Covariates	value ± standard error	t-value	p-value
COVID- 19 exposure	**− **0.737 ± 0.449	**− **1.639	0.101
Presence of siblings	0.588 ± 0.382	1.542	0.123
Night Awakenings			
Order Significant Covariates	value ± standard error	t-value	p-value
**Parents in remote working**	**− 1.034 ± 0.389**	**− 2.656**	**0.008**
	**Parents working from home negatively impacts Night awakenings**		
**Outdoor space**	**0.840 ± 0.381**	**2.205**	**0.027**
	**The presence of an outdoor space positively impacts Night awakenings**		
Physical Activity			
Order Significant Covariates	value ± standard error	t-value	p-value
Outdoor space	16.889 ± 908.488	0.019	0.985
Sex	0.675 ± 0.391	1.726	0.084
Information About Virus			
Order Significant Covariates	value ± standard error	t-value	p-value
**COVID-19 cases inside family**	**− 2.589 ± 1.130**	**− 2.291**	**0.022**
	**Who had COVID-19 cases inside family asks abnormally about virus**		
**ASD**	**− 1.525 ± *0.415***	**− 3.674**	**p < 0.001**
	**ASD children ask abnormally about the virus**		
**Outdoor space**	**0.965 ± *0.331***	**2.911**	**p = 0.004**
	**Children without an outdoor space ask abnormally about COVID-19**		
COVID-19 exposure	0.757 ± *0.519*	1.460	p = 0.144
Parents working from home	0.624 ± *0.324*	1.928	p = 0.054

Significant effects are highlighted in bold

### Descriptive Analysis of Clinical Data on the ASD Group

As shown in Fig. 2, before the lockdown, most subjects (94%) carried out rehabilitation therapies such as occupational and speech therapy, cognitive-behavioral therapy, and psychoeducational therapy. Slightly more than half (52%) maintained alternative therapies (remote therapy) during the lockdown. Approximately 30% of the children with ASD in the sample were placed in residential/semi-residential facilities before the lockdown, most of whom (70%) continued to receive treatment remotely. In addition, before the pandemic, 10 out of 35 children had a home educational intervention; 60% of these children maintained this intervention remotely. With regard to drug therapy, the analysis recorded a single case undergoing drug therapy before the lockdown. The therapy consisted of the intake of a neuroleptic, but the dosage was not changed during the lockdown. As shown in Fig. 1, a total of 43% of parents of children with ASD judged their child’s general symptoms to be worsened; another 43% of parents said they were stable; in contrast, a small percentage (14%) judged their child’s symptoms as improved during the lockdown. In terms of clinical symptoms, our data show a worsening, particularly of repetitive movements or speech patterns and restricted interests, in addition to a worsening of aggressive behavior among children who already had it before the pandemic. The alterations of clinical features in subjects with ASD are shown in Fig. 1. Of the sample, 57% of showed a regression of the skills previously acquired after the interruption of rehabilitation therapies during the lockdown. Finally, 37% of the children required a medical certification to justify a short exit from home to reduce anxiety and hyperactivity caused by persistent confinement at home and to prevent deregulatory behavior.

## Discussion

The present research was undertaken to study how the first COVID-19 lockdown in Italy impacted the everyday lives of young children from 3 to 6 years of age, studying the effects on distance learning activities, behavioral manifestations, routine changes, and virus-related anxiety. We focused on the weight of ASD condition on these impacts and we then discussed the specific trend of its signs and symptoms. A significant impact of ASD with regard to *Remote learning, Behavior changes*, and *Information about the virus* was found, whilst *Home activities*, *Sleep habits, Night awakenings* and *Physical activity* changed in all the young children of the sample, regardless of the presence of an ASD condition.

### Education and Learning

These results suggest that the presence of autism had a strong negative effect on learning: children with ASD had more difficulties with kindergarten distance learning. This was characterized by watching videos or listening to recorded audios, participating in video calls with educators, and classmates and carrying out certain educational home activities proposed by teachers. For many educators, as well as for tutors, the pandemic was the very first chance to propose remote activities to children, most of whom were not specifically trained to apply these methods. This could have contributed to amplifying the children’s difficulties, especially in those with ASD, who require individualized educational plans that remote learning could not completely provide. Moreover, the sudden necessity of remote programs could have contributed to logistic-related problems, such as the absence of a good internet connection or proper tools, given that a preschool child does not typically engage in online activities and families with young children could also be less prone to do so. Our findings are supported by studies that show difficulties in attending remote activities by children with special needs, who face barriers due to the absence of accessible materials and of the proper support to follow online programs (United Nations, 2020).

### Behavior and Emotion Changes in ASD

During the first wave of COVID-19, there were few confirmed cases of infection in toddlers or children. Even amongst children who contracted the disease, its course was mild to moderate, especially for those under the age of 5 (Bhuiyan et al., 2021), and the mortality rate was considerably low (Spaull, 2020). However, considering the overall implications of the pandemic, a strong impact on the mental health of young children was noticeable. Behavioral differences with typically developing children was so marked that it was necessary to build separate models for the two populations. The upset of usual activities indeed led children with ASD to a loss of balance, since one of the core domains of the spectrum is the inflexible adherence to routine (American Psychiatric Association, 2013). The presence of ASD also affected children’s behavior and emotions, as evidenced by the reports of parents: tolerance of frustration, aggressiveness, and hyperactivity worsened significantly. Numerous studies have found that prolonged isolation is a risk factor for functional decline and tends to fuel negative feelings such as loneliness, intolerance, irritability, anger, and problematic behaviors (Jacques et al., 2022). These exacerbations could have been increased by new routines practiced during home confinement, such as varying domestic activities, spending a great deal of time using computers, watching television or playing video games, which altered the usual dietary habits and sleep–wake rhythm (SIDiN, 2020). In addition, the alterations in sleep–wake rhythm could contribute to enhancing behavioral signs, as well as in worsening daytime functioning (Mazurek et al., 2019), given also the fact that most ASD children between the ages of 4 to 6 years suffer from insomnia (Posar & Visconti, 2020) and other sleep disturbances that are often associated with aggressive behaviors (Wang et al., 2021). According to evidence about the lockdown, parents reported mental health difficulties and increases in child behavioral problems since the onset of the pandemic (Yoshikawa et al., 2020); children with ASD were particularly vulnerable, considering (i) the specific support they needed in this period to preserve their psychological well-being (Colizzi et al., 2020) and (ii) the atypical emotion regulation that characterizes children with autism at all ages (Mazefsky, 2015). As a future development, it could be of interest to investigate to what extent poor non-verbal communication abilities led to frustration and thus affected the other variables of the study. Indeed, non-verbal communication is moderately correlated with *Behavior* (Spearman’s rho 0.58, p < 0.001), and its effect may transcend COVID-related modifications.

The presence of ASD also negatively influenced the proper requests for information about the virus. In this study, assessing whether children were asking for information about COVID-19 was intended to indirectly measure the children’s concerns and stress levels caused by the virus and by their state of health. Children with ASD did not ask for information about the disease; the Controls, however, showed a proper level of concern and anxiety about it. This could be elucidated by the marked difficulties for children with ASD concerning social reciprocity and communication (American Psychiatric Association, 2013) and also by their low proneness to understanding the hygiene-related guidelines needed during the pandemic (Mutluer et al., 2020). On the other hand, it should be acknowledged that children in the ASD group were seldom exposed to the virus (only 1 case out of 35), which justifies the lack of interest in the topic.

### Meaningful Effects in Preschool Children with and without ASD

The dependent variables that changed in all subjects irrespective of ASD condition, were influenced by the following main factors (i.e. independent variables). Having an outdoor space at home had a positive effect on sleeping, behavior, and virus-related anxiety in all children: fewer night awakenings, behavioral difficulties and concerns about the virus occurred in those who had a terrace or a garden. These results can straightforwardly be explained considering that outdoor activities have a positive impact on sleep and sedentary behavior in children under 5 years of age (World Health Organization, WHO 2019); outdoor activities, by reducing sedentariness, ensure a good quality of sleep in young children and thus can improve school performance as well (Janssen and Leblanc, 2010). Likewise, outdoor spaces in kindergarten are dominant for children’s activities: a natural environment captures their attention and thus has a positive influence on their psycho-physiological status (Lan & Guo, 2021; Biørgen, 2016).

### Everyday Life and Clinical Implications

As for the descriptive analysis of clinical data of children with ASD, results showed that telematic rehabilitation therapies were ensured in half of the cases during the first lockdown. Furthermore, the need for training of previously learned skills in children with ASD is clear-cut, as evidenced by the fact that almost half the parents of those who kept the telematic rehabilitation described a waning of acquired abilities as well as a worsening of their children’s general symptoms. With regard to symptoms, stereotyped movements and speech, restricted interests, and externalizing behaviors (hyperactivity, aggressivity) displayed an increase, probably as a consequence of the lack of scheduled activities and the frustration induced by home confinement. Concerning social and neurocognitive decline, the regression of previously acquired skills was arguably magnified by the disruption of clinical and educational routine, considering the ubiquitous limitation of usual pleasant activities, as well as of relationships with therapists, teachers, and tutors (Neece et al., 2020). Children with ASD in particular can experience a waning of acquired skills, a phenomenon known as *late regression* that can simulate *global delay* (Persico, 2018) and that is strongly influenced by environmental stimuli, i.e., by the possibility of constant training. Evidence about telehealth in toddlers with ASD underlines its strength in the chance to actually observe the home environments and, as a consequence, give families strategies that they could easily implement in daily activities. Indeed, even though families receiving in-person services report a slightly higher perceived improvement than those receiving tele-consults only, no significant differences were experienced between hybrid and in-person service models (Corona et al., 2021). Researchers offer two important statements concerning the use of mixed tools (i.e. technology-based and in-person tools) for managing ASD support: first, technology-based interventions in ASD subjects show a good effect if applied for a proper duration (Grynszpan et al., 2014); second, naturalistic developmental behavioral interventions proved to be effective in improving social engagement and cognitive abilities (Tiede & Walton, 2019). Finally, only one autistic subject in our sample was undergoing drug therapy and the dosage did not change during the lockdown. This is in line with the absence of specific drugs for treating the nuclear symptoms of autism (Sanchack & Thomas, 2016) and emphasizes once more the importance of environmental support in managing ASD.

## Limitations

A first limitation of this study could be the treatment of ASD as a single group, without sorting out children by severity and by level of support required. Although our single group analysis ensured the *spectrum* approach, it may be useful in the future to distinguish different levels of ASD severity, as assessed before lockdown, and to evaluate their different weights on the same variables. A second limitation is that little is known about the long-term effects of epidemics and quarantines on the mental health of children because most studies in the literature focus on anxiety symptoms in older age ranges (Brooks et al., 2020). It is therefore desirable to monitor the long-term mental health status of young people and to study how and to what degree the prolonged or discontinuous closure of schools, social isolation measures, and the pandemic itself affect children’s psychological well-being. A future challenge could be the follow up on the participants of the present study during the years of primary school, at least concerning the onset of possible new anxiety symptoms and clinical/behavioral trends caused by the years of pandemic.

## Conclusions

The first COVID-19 lockdown had a significant impact on preschool children with ASD, who showed learning, emotional, and behavioral difficulties. Through this study, it became clear how important it is to better manage children with this disorder during forced home confinement, especially by setting a routine similar to the one before lockdown. Close collaboration between parents and teachers is necessary to guarantee complete support to young children with special needs, who are often not autonomous in managing remote learning tools. In addition, clinicians and therapists should ensure care continuity using telemedicine when necessary, such as when home confinement is imposed for long periods, a circumstance that may occur again in the future. An ideal assistance for supporting children with ASD should indeed include a mix of technological and in-person activities to get optimal results, and the new approach to learning developed during the lockdown may provide a good opportunity for their ongoing use in the future.

## Supplementary Information

Below is the link to the electronic supplementary material.

Supplementary file1 (DOCX 28 kb)
