# Supplementary material for: Autism in Preschool-Aged Children: The Effects of COVID-19 Lockdown
Source: J Autism Dev Disord. 2023 Aug 4;54(10):3657–69. doi: 10.1007/s10803-023-06078-4 (PMC11461779; doi:10.1007/s10803-023-06078-4)
Supplement: Supplementary file 1 — Supplementary file1 (DOCX 28 kb) [file 10803_2023_6078_MOESM1_ESM.docx]

**The effects of Covid-19 quarantine in children attending Kindergarten**

The following questionnaire was created for a scientific study which aims to evaluate how children attending kindergarten are reacting to stress and lifestyle changes secondary to the lockdown. The questionnaire requires approximately 20 minutes to complete. Questions were chosen by the Child Neuropsychiatry Department of the Filippo Del Ponte Hospital (Varese) in collaboration with the University of Insubria (Varese). The questionnaire is totally anonymous and all the data provided will be sent and collected in anonymised form. It includes questions to be filled in by parents. By completing the questionnaire, the parent declares to have understood the purposes of this study and agrees to participate in it by providing the anonymous data necessary for its implementation.

1. **I take note of the above information, I declare that I understand the purposes of the questionnaire and I agree to participate in this survey**

- Yes
- No

*Sample characterization (independent variables)*

1. **Date of birth (child):**
2. **Sex (child):**

- Male
- Female

1. **Does your child have any of the following diagnoses? (it is possible to indicate more than one answer):**

- Language Disorder
- Attention Deficit / Hyperactivity Disorder (ADHD)
- Motor coordination disorder
- Oppositional-Provocative Disorder
- Obsessive Compulsive Disorder
- Epilepsy
- Autism Spectrum Disorder
- Tic
- None
- Other

1. **Attended class:**

- 1st year kindergarten
- 2nd year kindergarten
- 3rd year kindergarten

1. **Mother’s educational level:**

- Primary school education
- Middle school license
- Professional qualification (2-4 years)
- High school license
- Bachelor degree
- Master degree
- PhD or postgraduate specialization

1. **Father’s educational level:**

- Primary school education
- Middle school license
- Professional qualification (2-4 years)
- High school license
- Bachelor degree
- Master degree
- PhD or postgraduate specialization

1. **Mother’s occupation:**

- Precarious jobs, day laborers, housewives (canteen workers, waiters, babysitter, maid)
- Unqualified jobs, service jobs (school collaborators, social and health workers, doormen, bricklayers, tilers, warehouse workers, stokers, excavators, welders, cutters)
- Machine operators, semi-skilled jobs (workers, body builders, mechanics, shop assistants, cashiers, tailors, gardeners, painters, carpenters)
- Skilled manual labor, craftsmen, police and firefighters, conscripts and precarious and unskilled office workers (plumbers, electricians, carpenters, restorers, lithographers, hairdressers, dental technicians, dental hygienists, auxiliary nurses, hearing care professionals, security guards, cooks)
- Traders, clerics, owners of small businesses or farms (representatives, real estate agents, brokers, antique dealers, agricultural entrepreneurs)
- Technicians, semi-professionals, supervisors (educators, professional nurses, physiotherapists, instrumentalists, laboratory technicians, surveyors, graphic designers, technical employees)
- Small commercial business owners, industry owners, managers of low level, wage workers, primary school teachers (office workers administrators, accountants, entrepreneurs, freelancers and managers with educational qualifications undergraduate, journalists, librarians, translators, financiers)
- Mid-level managers, professionals, owners of medium-sized businesses, military officers (computer scientists, psychologists, graduate freelancers, managers, middle managers, pilots, councilors, public administrators, publishers, pharmacists, teachers in the secondary school)
- Senior managers, owners of large companies, professionals (professors and university researchers, ministerial politicians, doctors, lawyers, notaries, veterinarians, architects, engineers, physicists, accountants, geologists

1. **Father’s occupation:**

- Precarious jobs, day laborers, housewives (canteen workers, waiters, babysitter, maid)
- Unqualified jobs, service jobs (school collaborators, social and health workers, doormen, bricklayers, tilers, warehouse workers, stokers, excavators, welders, cutters)
- Machine operators, semi-skilled jobs (workers, body builders, mechanics, shop assistants, cashiers, tailors, gardeners, painters, carpenters)
- Skilled manual labor, craftsmen, police and firefighters, conscripts and precarious and unskilled office workers (plumbers, electricians, carpenters, restorers, lithographers, hairdressers, dental technicians, dental hygienists, auxiliary nurses, hearing care professionals, security guards, cooks)
- Traders, clerics, owners of small businesses or farms (representatives, real estate agents, brokers, antique dealers, agricultural entrepreneurs)
- Technicians, semi-professionals, supervisors (educators, professional nurses, physiotherapists, instrumentalists, laboratory technicians, surveyors, graphic designers, technical employees)
- Small commercial business owners, industry owners, managers of low level, wage workers, primary school teachers (office workers administrators, accountants, entrepreneurs, freelancers and managers with educational qualifications undergraduate, journalists, librarians, translators, financiers)
- Mid-level managers, professionals, owners of medium-sized businesses, military officers (computer scientists, psychologists, graduate freelancers, managers, middle managers, pilots, councilors, public administrators, publishers, pharmacists, teachers in the secondary school)
- Senior managers, owners of large companies, professionals (professors and university researchers, ministerial politicians, doctors, lawyers, notaries, veterinarians, architects, engineers, physicists, accountants, geologists)

1. **Family status:**

- Married parents
- Cohabiting parents
- Separated / divorced parents
- Uniparental nucleus

1. **Your child:**

- Is your child an only child?
- Does your child have a brother / sister?
- Does your child have more than one brother / sister?

1. **Is there a terrace/garden in your home?**

- Yes
- No

1. **Has anyone in your family been diagnosed with Covid-19?**

- Yes
- No

1. **Have you had contact with people positive for covid-19?**

- Yes
- No

1. **Are you a healthcare professional?**

- Yes (one)
- Yes (both)
- No

1. **Are you working at home?**

- Yes (one)
- Yes (both)
- No

*Dependent variables*

1. **Did your child have tutoring support before quarantine?**

- Yes
- No

1. **Does the tutor continue to carry out online activities?**

- Yes
- No

1. **Does your child attend remote educational activities?**

- Yes
- No

1. **Does your child ask for school re-opening?**

- Often
- Sometime
- Never

1. **How do you judge the global trend of your child's symptoms during the quarantine?**
   - Stable
   - Improved
   - Worsened
2. **Your child's social interaction during the quarantine is:**

- Stable
- Improved
- Worsened

1. **Tolerance to frustration during the quarantine is:**

- Stable
- Improved
- Decreased
- Never presented

1. **Aggressive/self-injurious behaviors are:**

- Stable
- Improved
- Worsened
- Never presented

1. **Hyperactivity during the quarantine is:**

- Stable
- Improved
- Worsened
- Never presented

1. **Are you organizing home-based activities alternative to school during the quarantine?**

- Yes
- No

1. **Your child's physical activity during the quarantine is:**

- Interrupted
- Unchanged
- Diminished
- Increased
- Never done

1. **During quarantine your child:**

- goes to sleep at the same time
- goes to sleep later
- goes to sleep earlier
- wakes up later
- wakes up earlier

1. **Night awakenings during quarantine are:**

- Unchanged
- Diminished
- Increased
- Never presented

1. **After hearing about Covid-19, does your child ask you about it?**

- Yes, often
- Yes, sometimes
- Never

*Questions specific to ASD children*

1. **As a result of the symptoms related to the autism spectrum, does your child take drugs?**

- Yes
- No

1. **If yes, does your child take neuroleptics?**

- Yes
- No

1. **How was neuroleptic therapy changed after school closed?**

- Increased
- Decreased
- Suspended
- Unchanged

1. **If yes, does your child take methylphenidate?**

- Yes
- No

1. **How was methylphenidate therapy changed after school closed?**

- Increased
- Decreased
- Suspended
- Unchenged

1. **If yes, does your child take benzodiazepines?**

- Yes
- No

1. **How was benzodiazepines therapy changed after school closed?**

- Increased
- Decreased
- Suspended
- No changes

1. **Your child's non-verbal communication during the quarantine is:**

- Stable
- Improved
- Worsened

1. **Echolalias/stereotypies during the quarantine are:**

- Stable
- Improved
- Worsened
- Never presented

1. **Hypo/hypersensitivity during the quarantine is:**

- Stable
- Improved
- Decreased
- Never presented

1. **Attentive skills are:**

- Stable
- Improved
- Worsened

**42. Repetitive behaviour during the quarantine are:**

- Stable
- Improved
- Decreased
- Never presented

**43. Did your child take melatonin before quarantine?**

- Yes
- No

**44. If yes, how was the melatonin dose changed during quarantine?**

- Increased
- Decreased
- Suspended
- Unchanged

**45. Did your child attend rehabilitation therapy before quarantine ?**

- Yes
- No

**46. Did your child continue to do rehabilitation therapy even during quarantine?**

- No
- Yes, no changes
- Yes, in alternative ways (e.g. online)

**47. Was your child attending a Rehabilitation Center before quarantine?**

- Yes
- No

**48. Has your child continued to attend the Rehabilitation Center during the quarantine?**

- No
- Yes, no changes
- Yes, in alternative ways (e.g. online)

**49. Did you need a specialist certification for the symptoms presented by your child during the quarantine?**

- Yes
- No

**50. The interruption of rehabilitation therapies has caused a worsening of your child's symptoms?**

- Yes
- No
